# In Silico Characterization of African Swine Fever Virus Nucleoprotein p10 Interaction with DNA

**DOI:** 10.3390/v14112348

**Published:** 2022-10-25

**Authors:** Claudia Istrate, Jéssica Marques, Pedro Bule, Sílvia Correia, Frederico Aires-da-Silva, Marlene Duarte, Ana Luísa Reis, Miguel Machuqueiro, Alexandre Leitão, Bruno L. Victor

**Affiliations:** 1 CIISA—Centre for Interdisciplinary Research in Animal Health, Faculty of Veterinary Medicine, University of Lisbon, 1300-477 Lisboa, Portugal; 2 Associate Laboratory for Animal and Veterinary Sciences (AL4AnimalS), 1300-477 Lisboa, Portugal; 3 BioISI—Biosystems & Integrative Sciences Institute, Faculty of Sciences, University of Lisbon, 1749-016 Lisboa, Portugal; 4 The Pirbright Institute, Ash Road, Pirbright, Surrey GU24 0NF, UK

**Keywords:** African swine fever virus, p10 protein, molecular dynamics, DNA binding function, K78R

## Abstract

African swine fever virus (ASFV) is the etiological agent of a highly contagious, hemorrhagic infectious swine disease, with a tremendous sanitary and economic impact on a global scale. Currently, there are no globally available vaccines or treatments. The p10 protein, a structural nucleoprotein encoded by ASFV, has been previously described as capable of binding double-stranded DNA (dsDNA), which may have implications for viral replication. However, the molecular mechanism that governs this interaction is still unknown, mostly due to the lack of a structural model for this protein. In this work, we have generated an ab initio model of the p10 protein and performed extensive structural characterization, using molecular dynamics simulations to identify the motifs and residues regulating DNA recognition. The helix-turn-helix motif identified at the C-terminal region of the protein was shown to be crucial to the dsDNA-binding efficiency. As with other DNA-binding proteins, two distinct serine and lysine-rich regions found in the two helices were identified as key players in the binding to DNA, whose importance was later validated using experimental binding assays. Altogether, these findings may contribute to a better understanding of the p10 function in ASFV replication.

## 1. Introduction

African swine fever (ASF) is one of the most severe viral diseases in swine (domestic and wild). It is a highly contagious and devastating hemorrhagic disease with a tremendous sanitary and economic impact, notifiable to the World Organization for Animal Health (WOAH), and is considered a major transboundary animal disease by the Food and Agriculture Organization (FAO). It represents one of the greatest global threats to the swine industry, including the international trade of live animals and pork products.

Described for the first time in Kenya in 1921 [1], ASF is considered endemic in most countries of sub-Saharan Africa. In 1957 and 1960, ASF was introduced to Portugal and spread to neighboring European countries, South America, and the Caribbean Islands [2]. From this incursion outside Africa, only the island of Sardinia remains infected. However, in 2007, ASF was introduced in the Caucasus region and spread to Central–Eastern Europe and Asia (including China), where outbreaks are still ongoing [3]. More recently, outbreaks were also registered in the Dominican Republic and Haiti, thus reaching the American continent [4]. The exponential growth and spread of ASF have transformed this disease into an acute and global animal health emergency.

The major outbreaks that are currently ongoing in Eastern Europe and Asia, together with the eminent global spread of the virus, accentuate the need for additional efforts to improve the scientific knowledge of the fundamental biology of the ASF virus (ASFV). The complexity of the virus and limitations in the current understanding of its interaction with the host, together with the lack of correlates of protection, can explain the fact that vaccine development is still in its infancy. Currently, prevention, control, and eradication methods are limited to early diagnosis, quarantine, and the elimination of susceptible animals in most of the affected areas; thus, the demand for an efficient, safe, and widely available vaccine is very high. The most promising strategies to develop vaccines for this type of virus have been focused on the use of modified live viruses derived through gene deletion approaches. However, to ensure that this approach is efficient, scientists should focus on protein targets with high genetic stability to avoid escaping without phenotypic costs. Therefore, a deep understanding of the life cycle of the ASF virus, and accurate identification and characterization of key proteins involved in such functions, will certainly increase the success of vaccine development to prevent this viral infection.

ASFV is a large, enveloped, linear, double-stranded (ds) DNA virus, the single member of the family *Asfarviridae*, being the only described DNA arbovirus. The ASFV genome ranges from 170 to 190 kbp and contains (depending on the isolate) between 150 and 167 open reading frames (ORFs), many of them encoding proteins involved in nucleotide metabolism, transcription, replication, and repair [5]. The protein p10, encoded by ORF K78R, is one of the 68 ASFV structural proteins [6] and is putatively involved in ASFV replication. Furthermore, p10 was identified in bush pigs and in domestic swine that have recovered from ASFV infection as one of the principal serological immunodeterminants [7].

The p10 structural protein is localized in the viral nucleoid and has been shown to exhibit DNA-binding activities, having a role in the assembly of the DNA-containing nucleoid [8,9]. During viral infection, p10 exhibited nuclear import capacity and accumulation in the nucleus [9], which correlates well with the previous description showing that this protein can bind single- and double-stranded DNA [8]. However, the way in which this interaction/binding process occurs is still unknown. Nucleoprotein p10 is a small 78-amino-acid protein, with high content of serine and lysine residues. Several of these serine residues are found in serine-rich regions [8], characteristic of some nucleic-acid-binding proteins. Moreover, the lysine/arginine sequence found between residues 71 and 77, previously described to be important for the active nuclear import of the p10 protein [9], was also shown to be relevant to the DNA-binding efficiency in several other DNA-binding proteins [10].

To identify the structural regions of ASFV p10 responsible for the binding to DNA, we initiated this project by generating a full and functionally relevant, truncated representative 3D model of the protein. After identifying the most important structural features of these models, we performed long molecular dynamics (MD) simulations in the presence of dsDNA. With these simulations, we pinpointed the key residue players in the interaction process with the dsDNA and used this information to generate several p10 mutants and validate their role in this binding process. Finally, the binding affinity of the wild-type (wt) p10 and the generated mutants was experimentally evaluated using ELISA assays, confirming most of the initial computational predictions.

Overall, with this work, we have gathered crucial structural information on the p10 nucleoprotein and its interaction with dsDNA, which will be of the utmost importance in future developments of vaccines for ASFV.

## 2. Materials and Methods

### 2.1. Model Generation of wt:p10

To generate a three-dimensional (3D) model of the wild-type nucleoprotein p10 (wt:p10) from ASFV, we have used the Robetta webserver [11]. In this server, if a confident match to a protein with a known structure is found at the PDB databank, a comparative modeling approach will be used. Otherwise, if no match is found, the prediction of the structure is performed using the de novo approach implemented in the server based on the fragment insertion method [11]. Since the sequence homology between wt:p10 and currently available protein structures at the PDB databank [12] is lower than 20%, the latter approach was used to generate structural models of a full protein sequence (78 a.a.) and a truncated version (43 a.a.) of wt:p10.

### 2.2. Molecular Dynamics Simulations

All molecular mechanics/molecular dynamics (MM/MD) simulations were performed using the GROMACS 2018.6 software package [13] and the OL15 refinement of the Amber14sb force field [14,15,16].

The 3D models of the p10 proteins were used to build simulation systems with the single wt:p10 protein (78 residues full length protein and 43 truncated). Additionally, systems composed of wt:p10, and some of its mutants, in the presence of a dsDNA strand (21 base pairs, from PDB ID: 1du0 [17]), were also built and simulated. The MD simulations of the apo p10 systems and complexed with dsDNA were performed in 3 replicates of 100 ns and 1 microsecond (μs), respectively. Moreover, in all dsDNA mutant complex simulations, we used 8 replicates of 1 microsecond. All systems were initially solvated using TIP3P water molecules [18,19] in a dodecahedric box, and the overall system charge was neutralized by randomly replacing water molecules with the necessary sodium ions, via the GROMACS genion tool. The systems underwent a 2-step energy minimization procedure using the steepest descent algorithm [20]: first with no constraints, and second with constraints in covalent bonds involving H atoms. To initialize the system, NVT and NPT short MD segments (100 ps) were performed to generate velocities, according to a Maxwell distribution at 300 K (26.85 °C) of temperature, and to couple our systems to a specific isotropic pressure (1 bar). The particle mesh Ewald (PME) method was used to treat long-range electrostatic interactions [21,22], with a Fourier grid spacing of 0.12 nm and a cutoff of 1.4 nm set to define the direct contributions. Lennard–Jones interactions were calculated using a neighbor pair list with a cutoff of 1.4 nm and using a Verlet scheme [23]. p10 and dsDNA bonds were constrained using the parallel linear constraint solver P-LINCS [24], while water molecules were constrained using the SETTLE algorithm [25]. The temperature was kept at 300 K (26.85 °C) using a Nosé–Hoover thermostat [26,27] with a coupling constant of 1 ps, and an isotropic Parrinello–Rahman barostat [28,29] was used to keep the pressure constant at 1 bar, with a coupling constant of 5 ps and compressibility of 4.5 × 10^−5^ bar^−1^.

Analyses were performed using several GROMACS tools [13]. In addition, plots were generated using Gnuplot [30] and all 3D representations of the simulated systems were rendered using PyMOL [31]. The representative structures presented in this work were obtained by applying a cross-RMSD protocol (an RMSD matrix) where a central structure is identified as the conformation that minimizes the sum of RMSD values calculated against the remaining ones [32].

### 2.3. MM/PBSA Calculations

The binding free energies of wt:p10 and its mutants in complex with the dsDNA molecule were calculated using the program g_mmpbsa, developed by Kumari et al. [33,34]. Similarly to other studies [35], we have used a single trajectory approach, which assumes that the conformational spaces of the p10 protein and dsDNA in the bound and unbound states are identical. This is a rough approximation that will lead to large deviations in the estimation of the absolute binding energies. However, we are interested mainly in the relative free binding energies, focused on energy differences between binding modes of the same system or between two systems that differ in a few mutations. In this case, the single trajectory approach is adequate and allows us to discard the calculation of the elusive entropic contribution to the free energy. In each calculation, 500 snapshots of each system were used to calculate the final binding free energies (one snapshot per nanosecond taken from the equilibrated parts of the simulations—between 500 and 1000 nanoseconds). The final equation used to calculate the binding free energy ($\Delta G_{bind}$) showcases the different contributions and is expressed as

(1) $$\Delta G_{bind}=\Delta G^{vdW}+\Delta G^{ele}+\Delta G^{polar\;solvation}+\Delta G^{nonpolar\;solvation}$$

with $\Delta G^{vdW}$ and $\Delta G^{ele}$ being the p10:dsDNA van der Waals and electrostatic energy changes in vacuum, respectively. The solvent contributions are decomposed as polar and nonpolar. Since this polar solvation energy is known to depend on the chosen value for the dielectric constant ε_solute_ of the complex [36], we evaluated two of the most used values (4 and 8) and concluded, independently of the chosen value, that no major impact on the final interpretation of the results was observed. Hence, we adopted ε_solute_ = 8 for the results shown in this work. The atomic radii parameters chosen were those from the GAFF force field [37].

To estimate the nonpolar solvation energy, we have used the fact that this contribution has a linear dependence on the solvent-accessible surface area (SASA), expressed as follows [38]:

(2) $$G^{nonpolar\;solvation}=\gamma_{surf}SASA+b$$

where *γ_surf_* is related to the surface tension of the solvent and whose value was set to 0.0226778 kJ/(mol A^2^), and where b is the offset fitting parameter, set to 3.84928 kJ/mol. A solvent probe radius of 0.14 nm was used to calculate the SASA.

### 2.4. Design of p10 Mutations

All designed mutants were built using the selection mutation python script from Modeller version 10.0 [39,40,41,42]. The last snapshot of one of the replicates of wt:p10 complexed to dsDNA MD simulations was used as a structural template to generate the desired mutations. The software considers this template to rebuild the coordinates of the original residues to match the desired target mutated amino acids.

### 2.5. p10 and Mutants’ Production and Purification

The p10-encoding K78R gene and selected mutants were synthesized, after codon optimization for *E. coli* expression, and cloned into the N-terminal His-tag fusion vector pET28a (Genscript, Leiden, The Netherlands). They were subsequently expressed in *E. coli* and purified by immobilized-metal affinity chromatography. Briefly, the recombinant gene plasmids were used to transform E. coli strain BL21 competent cells. The transformed bacteria were then selected using the appropriate antibiotic and grown in LB media at 310 K. After the bacteria reached the exponential growth phase, protein expression was induced by the addition of 1 mM IPTG and carried out overnight at 292 K. The cells were collected by centrifugation and re-suspended in HEPES buffer pH 7.5 containing 1 M NaCl and 10 mM imidazole. After cell lysis by sonication and subsequent centrifugation, the supernatant was injected into the HisTrap column (Cytiva, Marlborough, MA, USA) loaded with nickel. The unbound protein was washed out with 10 mM imidazole buffer, and the recombinant protein was eluted with increasing imidazole concentrations: 35 mM, 60 mM, and 300 mM. The collected fractions were selected according to SDS-PAGE analysis, pooled, and the buffer exchanged for 50 mM HEPES pH 7.5, 200 mM NaCl, 0.5 mM CaCl_2_ buffer.

### 2.6. Protein Quantification

After purification, the concentration of p10 and its mutant derivatives was determined by a commercial kit, the Coomassie (Bradford) Protein Assay (Thermo Scientific, Waltham, MA, USA), using the microplate format procedure. Here, 5 µL of the mutant derivative was pipetted into a 96-well plate (Thermo Scientific, Waltham, MA, USA). Coomassie reagent was added (250 µL/well) and it was mixed for 30 s. The plate was incubated at room temperature for 10 min, and then the absorbance was read at 595 nm wavelength with a Microplate ELISA Reader (Biorad, Basel, Switzerland). A standard curve was prepared using a range of known BSA concentrations. This standard calibration curve was used to estimate p10 and mutant derivatives’ protein concentrations (µg/mL).

### 2.7. ELISA Assays

Streptavidin-coated plates (Biomat, Trento, Italy) were blocked with blocking buffer (1% *w/v* BSA in PBS) for one hour at 310 K. After washing four times with washing buffer (0.05% Tween 20 in PBS), biotinylated oligos were added to plates (10pmol/well in blocking buffer). Plates were incubated for 1 h at 310 K and then washed again 3 times with washing buffer. The wt:p10 protein and/or mutants (M1–M8) at different concentrations (12.5, 6.25, 3.125, 1.56 µM) were added. Samples and standards were run in duplicate. The last line was used as a negative control and plates were incubated for one hour at 310 K. After washing 3 times, anti-histidine horseradish peroxidase (Sigma-Aldrich, St. Gallen, Switzerland, dil. 1:1000) was added, and plates were incubated for one hour at 310 K. After washing 3 times, freshly prepared ABTS (2,2’-azinobis [3-ethylbenzothiazoline-6-sulfonic acid]-diammonium salt) substrate solution (Sigma-Aldrich, St. Gallen, Switzerland) was added to each well. The plate was then incubated at room temperature in the dark. The ELISA plate was read at appropriate time points (5, 10, 30 min), at the optical density of 405–415 nm (using 495 nm as reference), using the Microplate ELISA Reader (Biorad, Basel, Switzerland).

### 2.8. Statistical Analysis

The statistical analysis was performed using GraphPad Prism8 software. Two-way ANOVA followed by Dunnett’s multiple comparison test was used to evaluate differences between the eight p10 mutants and the wt protein.

## 3. Results

### 3.1. wt:p10 Model Building and Its Characterization

**Figure 1:**
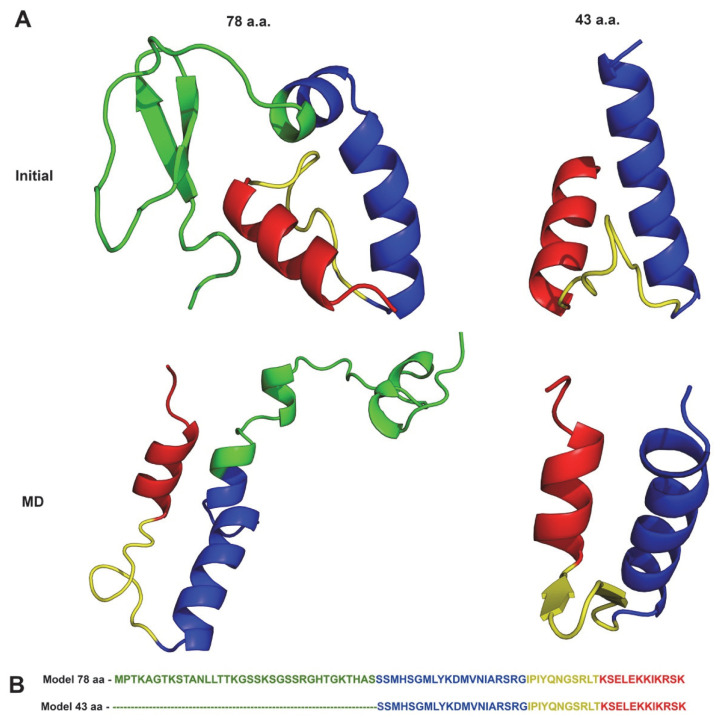
(**A**) Structural representation of the ab initio models (initial) and of the most representative (central) conformations obtained from MD simulations of wt:p10. (**B**) a.a. sequences of both models. The full and partial a.a. sequences of both models are colored with the same color scheme used in the structures.

Secondary structure predictions performed with the PsiPred webserver [43] show that the first 35 residues of the full-length wt:p10 have no regular secondary structure arrangement (Figure S1). From residue 36 onward, this protein is predicted to have a helix-turn-helix motif, similar to what was reported in other DNA-binding proteins [44]. Both the 78- and 43-amino-acid (a.a.) wt:p10 models generated with the Robetta webserver (Figure 1A) consistently show the presence of this structural motif in the same sequence of a.a. Interestingly, in this motif, it is also possible to observe the existence of a small misalignment of the two helices, which seems to be stabilized by several hydrophobic a.a. residues. To evaluate the dynamic stability of the reported motif, we performed MD simulations with both models. As shown in the C-alpha RMSD plots (Figure S2A,B), in both models in all three replicate simulations, after 50 ns of simulations, the C-terminal region of both models reached an equilibrated conformational state, which we have represented with their central structure (Figure 1A) [32]. In the simulations with the 78 a.a. model, the helix-turn-helix motif was slightly lost when compared to the truncated 43 a.a. wt:p10 model. The high conformational variability of the N-terminal region of this protein (Figure S2B,C), contributes to the observed destabilization of the C-terminal wt:p10 structural helix-turn-helix motif. Interestingly, these observations were not noted in the 43 a.a. model simulations, suggesting that the helix-turn-helix motif of wt:p10 is structurally stable. This observation, and the respective conservation of the misalignment between the two helices, suggests that the C-terminal region of the protein may be important in the DNA-binding function of wt:p10, similarly to other proteins containing helix-turn-helix motifs [44].

With the objective of identifying the structural determinants involved in the interaction of p10 with dsDNA, we focused our efforts on the 43 a.a. wt:p10 model in the subsequent modeling work (see Figure 1).

### 3.2. Prediction of the Interaction of the Truncated wt:p10 with dsDNA

**Figure 2:**
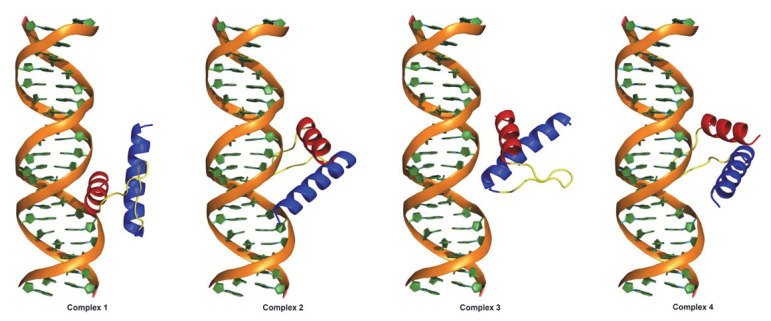
Starting configurations used in the wt:p10 MD simulations in complex with dsDNA.

Without any structural hints regarding the wt:p10 protein, or its complex with dsDNA, we used MD simulations to explore their configurational space. To minimize the user bias, we prepared four significantly different starting configurations of the wt:p10 model relative to a dsDNA molecule (Figure 2) and performed long MD simulations (3 × 1 µs) to study the complex formation and relaxation.

**Figure 3:**
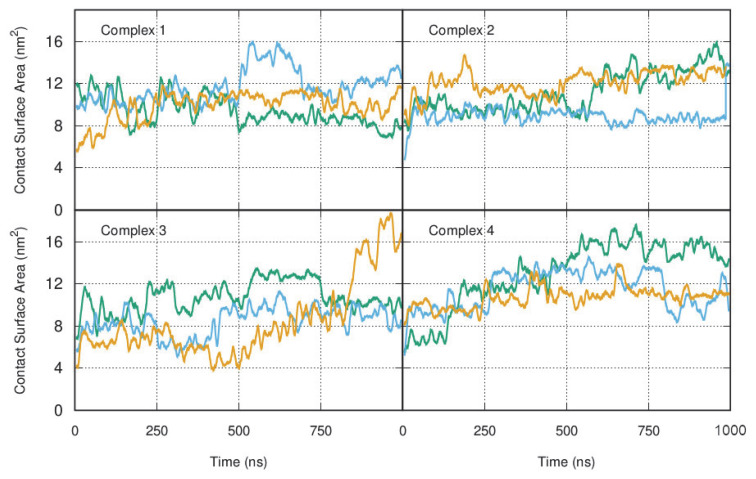
The contact surface area between wt:p10 and the dsDNA calculated for all MD simulations. Replicate 1, 2 and 3 from each simulated system is respectively colored in green, blue, and yellow.

**Figure 4:**
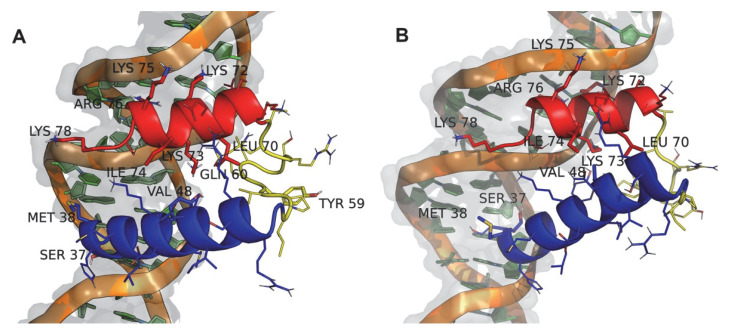
Representative structures of wt:p10/dsDNA from complex 2 (**A**) and 4 (**B**) systems. The wt:p10 protein is colored using the previously defined scheme (Figure 1). Residues from wt:p10 showing strong intramolecular and intermolecular interactions with dsDNA are represented as labeled thicker sticks.

The contact surface area variation for each dsDNA/wt:p10 complex shows an increase over time for most simulations, more evident after an equilibration period (Figure 3). After 500 ns, the conformations adopted by the wt:p10 protein relative to the dsDNA in these systems have higher surface complementarity, suggesting the formation of stable complexes (Figure S3). Analyzing these results and looking at the representative structures of the complexes in equilibrium, we identified the most important structural features of wt:p10 driving the formation of these stable arrangements (Figure 4). In the representative structures of complexes 2 and 4, selected for their high contact surface area, the helix-turn-helix motif and the previously observed misalignment of the two wt:p10 helices are kept in the interaction with dsDNA. Additionally, in both models, the N-terminal helix of the wt:p10 protein (colored in blue) directly interacts with the major groove of the DNA, while the C-terminal helix (colored in red) adopts a similar interaction profile in the minor groove. Focusing specifically at the N-terminal helix of the wt:p10 protein models, we observe that the first five residues show a deeper insertion in the major groove of the dsDNA, while the remaining residues seem to be more solvent-exposed. These first residues of the 43 a.a wt:p10 model form a serine-rich motif, also observed in other DNA-binding proteins, to direct the interaction with DNA [8]. The unstructured loop connecting both helices does not seem to interact with dsDNA, despite the presence of some positively charged residues. These residues prefer to be in well-solvated positions, without contributing to the stabilization of the observed complexes. Furthermore, the C-terminal helix is partially embedded in the minor groove of the dsDNA, with a very good surface/shape complementarity. Although smaller (only composed of 12 residues), the positively charged residues found in this helix establish stable electrostatic interactions with the negatively charged region of the dsDNA phosphate backbone. Lys-72, 73, 75, 78, and Arg-76 are the residues providing close interactions with the minor groove of the dsDNA, contributing to the apparent stability of these complexes (Figure 4).

**Figure 5:**
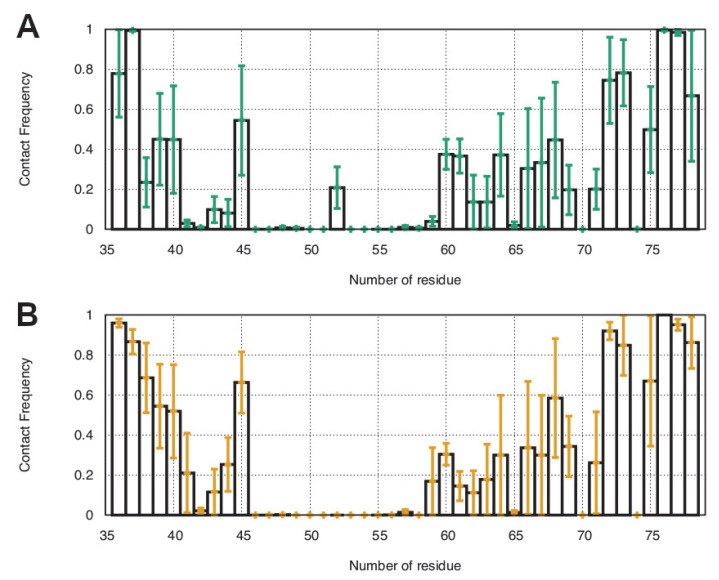
Contact frequency between each wt:p10 residue and the dsDNA in complexes 2 (**A**) and 4 (**B**). The standard errors of the mean between replicates were calculated and are highlighted with the green and yellow bars.

To identify the residues from wt:p10 that contribute the most to the complexes’ formation, we calculated the contact frequency per residue (Figure 5). On average, the first five residues of the N-terminal helix show a high number of contacts with the dsDNA. This agrees with the representative complex structure described in Figure 4, where Ser-36, Ser-37, Met-38, His-39, and Ser-40 were shown to be deeply inserted in the major groove of the dsDNA. Additionally, the final seven residues found in the wt:p10 C-terminal helix also establish a high number of contacts with the dsDNA. Correlating these observations with the representative configurations of the complexes (Figure 4), it becomes clear the important role of hydrogen bonds and ionic interactions between Lys-72, Lys-73, Lys-75, Arg-76, Ser-77, and Lys-78 and the phosphate backbone of the dsDNA delimiting the minor groove. Arg-76 is the residue more frequently interacting with the dsDNA, probably due to its side chain adopting a stable and persistent position embedded in the minor groove of the dsDNA (Figure 4). The arginine residue is particularly suitable for this role since, despite carrying a positive charge, it retains significant hydrophobicity in a planar group (guanidinium) to promote its side chain insertion deep into the dsDNA base pair region. The residues in the loop segment (60–69) also interact with the dsDNA, but these are less frequent and are probably transient in nature, due to the high flexibility of this wt:p10 region.

### 3.3. Design of p10 Mutants

We have developed several mutants of the p10 protein in complex with dsDNA to evaluate the role of these mutated residues in the interaction with dsDNA. As described in Table 1, M1–4 were prepared as single-point mutants. M1 and M2 were designed to evaluate the effect of introducing a positively charged residue in the loop region, while M3 and M4 were devised to evaluate the effect of adding, respectively, one negative and one positively charged residue at the C-terminal loop. Additionally, M5–8 were designed as triple mutations to evaluate the importance of the different wt:p10 structural motifs previously identified in dsDNA interaction. In M5, Val-48 from the N-terminal helix, and Leu-70 and Ile-74 from the C-terminal helix, were mutated to negatively charged aspartate residues. These three residues were identified in the apo wt:p10 and in the complex simulations to be in close proximity, establishing highly stable hydrophobic interactions, pivotal for the helix-turn-helix motif. By mutating them into charged aspartate residues, we will promote the strong destabilization of the interaction between the two helices in p10. This will allow us to study the impact of this disruption in the p10 overall fold and on p10′s interaction with dsDNA. Furthermore, mutations defined in M6 and M7 focused solely on the p10 C-terminal helix. In M6, three positively charged residues, previously shown to establish a high number of interactions with the dsDNA, were mutated to alanine residues. In contrast, in M7, the mutations were focused on the first three residues of the same p10 helix. While M6 was designed to evaluate the importance of the highly positive electrostatic potential found in this helix in the interaction with the negatively charged dsDNA backbone, M7 was outlined to determine how the first three residues of the C-terminal helix from p10 (showing a reasonable number of contacts with the dsDNA—see Figure 5) influence the interaction profile with dsDNA. Finally, M8 was built to evaluate the importance of the serine-rich region found at the N-terminal helix (previously described to be characteristic of DNA-binding proteins) in the interaction with dsDNA. By mutating Ser-36, Ser-37, and Ser-40 to aspartate residues, the objective was to substitute these polar residues by negatively charged a.a. to promote a destabilization in the interaction of this part of the p10 protein with dsDNA. The C-terminal 43 a.a. region used in the simulations is highly conserved and none of the produced mutations have been previously reported in an ASFV isolate (Figure S4), suggesting that they are unlikely to spontaneously occur.

### 3.4. In Silico Evaluation of the Truncated p10 Mutants and Their Interaction with DNA

**Figure 6:**
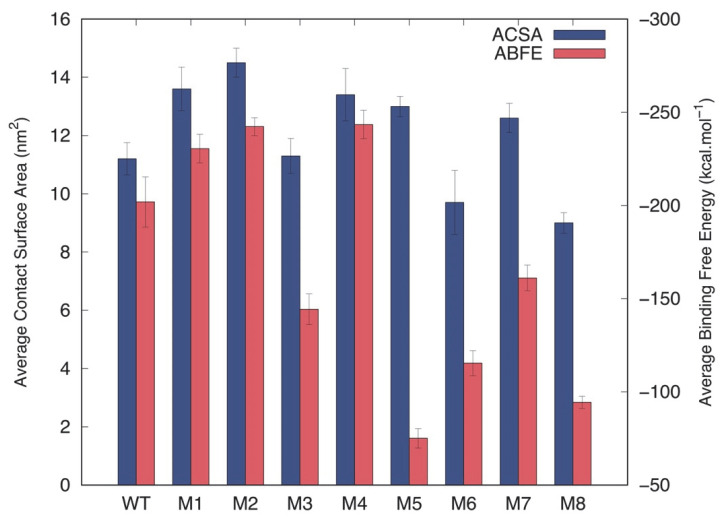
Average contact surface area (blue boxes) and average binding free energy (red boxes) between the wt:p10 and the evaluated mutants with the dsDNA.

To evaluate the effect of the previously described mutations on the dynamics of the p10 and dsDNA complex, we performed (8 × 1 µs) MD simulations of each system. All simulations from the studied p10:dsDNA complexes seem to have reached equilibrium in their contact surface area after 500 ns (Figure S5). From the calculated average values (Figure 6), we observe that M6 and M8 showed lower contact surface area values with the dsDNA. Furthermore, M1, M2, M4, M5, and M7 evidenced relevant increases in the contact surface areas, which could have an impact on the stabilization of these complexes when compared to the wt:p10 reference. The remaining M3 mutant showed similar average contact surface areas to the wt:p10 reference.

**Figure 7:**
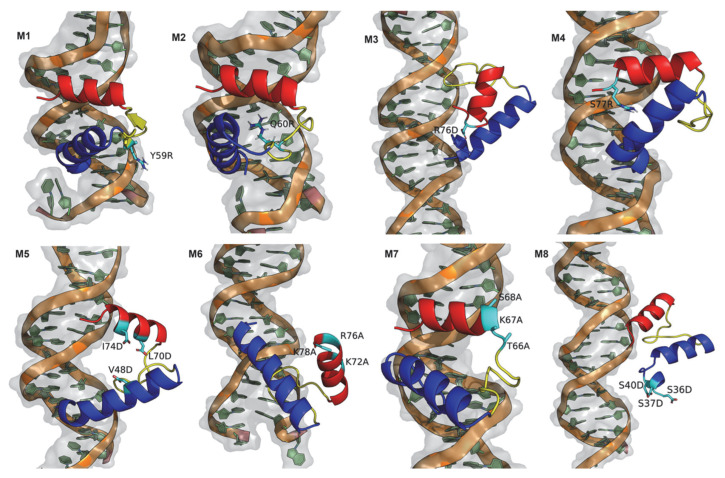
Representative structure from all evaluated mutation systems. p10 protein is colored according to the previously used color code: N-terminal helix in blue, the loop in yellow, and the C-terminal helix in red. The mutated labeled residues are identified as sticks, colored in cyan.

To correlate these results with detailed structural information, we selected a representative structure (the central structure—see Methods) for each mutant (Figure 7) and calculated the average binding free energies (ABFE) for the equilibrated parts of the simulations using the MM/PBSA method. Although M1 and M4 have very similar interaction configurations in respect to the ones observed for wt:p10 (Figure 4), these mutants show a lower ABFE, corresponding to more stabilized complexes (Figure 6—red bars). Despite the relatively high average surface area of contact with the dsDNA, the configurations for all the remaining mutants suggest significant structural rearrangements. In M2, the C-terminal helix interacts with the minor groove of the dsDNA, similarly to what is observed in wt:p10. However, the loop at which the mutated arginine residue is found moves towards the major groove to promote stable electrostatic interactions with the backbone of the DNA, pushing out the p10 N-terminal helix from the major groove cleft. Despite the dissimilarity of interaction in respect to the observed configurations with wt:p10, this mutant shows a lower ABFE, indicating that the observed adjustments provided more highly stabilized complexes. In M3, substituting a residue with the opposite electrostatic properties (R76D) led to a large rearrangement of the overall protein fold. The C-terminal helix was pushed out of the minor groove of the dsDNA, while the N-terminal helix moved away from the DNA major groove. As expected, the adopted configurations had a huge negative impact on the ABFE between this mutant protein and the dsDNA (Figure 6).

As previously mentioned, the M5 triple mutant was designed to destabilize the helix-turn-helix p10 motif and to determine its impact on the interaction with the dsDNA. The substitution of three hydrophobic residues with negatively charged aspartates led to the disruption of the characteristic hydrophobic zipper established between the two helices. The interaction pattern of both helices with the dsDNA was affected, and while the N-terminal helix remained at the dsDNA major groove, the C-terminal helix seemed to be pulled out of the native minor groove, with the most pronounced negative impact on the calculated ABFE (Figure 6). Regarding the M6 mutation, three positively charged residues at the C-terminal helix were mutated to the uncharged and hydrophobic alanine residues. As shown in Figure 7, both p10 helices moved away from the major and minor grooves of the dsDNA without losing the characteristic helix-turn-helix, which reduced the average contact surface area when compared to the wt:p10 complex configuration, and the consequent negative impact on the ABFE (Figure 6). In M7, the focus of the triple mutation was on the final part of the loop, close to the beginning of the C-terminal helix. The substitution of polar and charged residues by alanines imposed some changes on the position of the N-terminal helix of the p10 protein. The C-terminal helix remained closely bound to the dsDNA, similarly to what was observed for wt:p10. However, the N-terminal helix was displaced from the DNA major groove in a configuration that, although showing, on average, a higher contact surface area (Figure 6—blue bars), disrupted the typical helix-turn-helix motif, with a negative impact on the ABFE. Finally, in the M8 mutant, the residues in the serine-rich region located at the beginning of the N-terminal helix were mutated to the negatively charged aspartate residues. This triple mutant leads to the loss of both the helix-turn-helix p10 motif and the interaction of the N- and C-terminal helices with the major and minor grooves of the dsDNA (Figure 7). These significant structural changes induced a decrease of 25% in the average contact surface area of the p10 mutant with the dsDNA, and a reduction of 50% in the calculated ABFE, making it the second most perturbing mutation among the ones evaluated.

### 3.5. Experimental Evaluation of the Binding to DNA of the Mutated and Wild-Type p10

**Figure 8:**
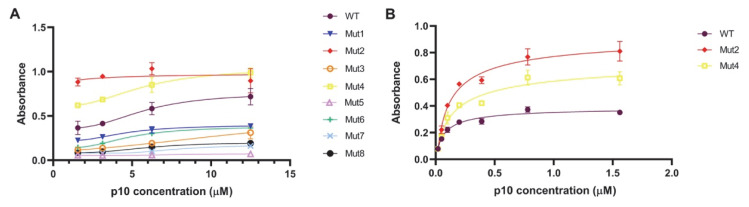
ELISA assay quantifying the dsDNA-binding affinity of M1–8 mutants (**A**) and at lower concentrations of M2 and M4 (**B**).

We have devised an ELISA assay to experimentally validate the dsDNA-binding effects observed in the computationally designed p10 mutants. As shown in Figure 8A, for the protein concentrations tested, only the M2 and M4 mutants showed increased binding to dsDNA compared to wt:p10 (*p* < 0.0001). Interestingly, in these mutants, the activity levels are similar in the concentrations and the binding does not seem to increase with increased protein concentrations. The remaining mutants bind less efficiently to dsDNA than wt:p10 (*p* < 0.0001).

An additional assay was performed at lower protein concentrations to confirm M2 and M4’s strong binding to dsDNA when compared to the wt:p10 protein (*p* < 0.0001) (Figure 8B). These results are in almost perfect agreement with our computational predictions. Only the M1 system seems to be less stable than calculated, indicating that our MM/PBSA protocol slightly overestimates the role of electrostatics, with the addition of an extra positive charge to the protein.

## 4. Discussion

ASF is a highly contagious viral disease that affects the members of the Suidae family. In the past few decades, this disease has had a catastrophic impact on the pig trade and production, with serious implications for global food security. Better knowledge of the basic virology and the structural proteins of ASFV would contribute considerably to advances in diagnostic tools and vaccine design. Taking into consideration these facts, in this work, we have performed a thorough structural characterization of the p10 protein, and, more importantly, we have identified its structural features regulating the interaction with dsDNA.

Since no three-dimensional structure of the wt:p10 protein is currently available and it shows very low sequence homology with available proteins in protein databases, we have used the Robetta server [11] to derive an initial structural model. Using long MD simulations, we have shown that only the globular part of this protein (the last 43 residues) shows significant structural motifs characteristic of DNA-binding proteins. We identified a DNA-binding helix-turn-helix motif that interacts in an apparently stable configuration with dsDNA. Starting from different unbiased initial configurations of the p10 protein relative to a dsDNA, we observed that the Lys-enriched C-terminal helix tends to interact with the dsDNA minor groove. Moreover, the N-terminal helix, rich in serine residues, was also identified to form highly stable interactions with the dsDNA major groove. These two misaligned helices are connected with a flexible loop, and this structural arrangement promotes a stable overall interaction configuration with the dsDNA. To further validate the importance of this p10 structural motif in the interaction with dsDNA, and the role of several of its residues, we performed single and triple mutations on this protein and evaluated their effects in the interaction with dsDNA. Our results indicate that both the C-terminal helix rich in lysine residues and the serine-rich residues found in the N-terminal helix of p10 are vital to the interaction with dsDNA. Additionally, we also observed that the characteristic helix-turn-helix structural motif is essential for the efficiency of the dsDNA binding. To validate the computational predictions, we prepared ELISA binding assays between p10 (wt and mutants) and dsDNA. From these experiments, we confirmed the predicted effects of most mutations in the interaction with dsDNA, highlighting the importance of the p10 helix-turn-helix structural motif and the identified serine- and lysine-rich regions.

Overall, our multidisciplinary approach pinpointed the role of electrostatics in the strength of the interactions between p10 and DNA. Additionally, it seems that more important than the number of cationic residues in p10 is the specific location where they are inserted/mutated.

The strong structural characterization of p10 performed in this work will open the door to further studies on the role of these amino acid residues in p10′s function during viral replication and assembly. The first step towards this will be the validation of our predictions by crystallography, followed by the introduction of these mutations in the virus genome itself. This would allow us to further understand the role of p10′s binding to DNA during the virus life cycle. We speculate that reduced binding to DNA may impact ASFV replication in vitro and result in virus attenuation in vivo, thus providing a rationale for the development of a live attenuated vaccine for ASF. Interestingly, p10 is recognized by antibodies produced by pigs that survive ASFV infection [7,45], indicating that the deletion of p10 may provide a target to develop a vaccine, allowing us to differentiate between infected and vaccinated animals (DIVA). Indeed, another ASFV-encoded DNA-binding protein was shown to be non-essential for virus replication in vitro but an important virulence factor in vivo [46], suggesting that this may also be the case for p10, warranting further studies on its function.

## Figures and Tables

**Table 1:** The 43 a.a. p10 mutant systems simulated in this work. The sequence of each mutant is colored according to the previously defined color code. Mutated residues are highlighted in cyan.

Reference System	Amino Acid Sequence
WT	**SSMHSGMLYKDMVNIARSRG** **IPIYQNGSRLT** **KSELEKKIKRSK**
M1—Y59R	**SSMHSGMLYKDMVNIARSRG** **IPI** **R** **QNGSRLT** **KSELEKKIKRSK**
M2—N60R	**SSMHSGMLYKDMVNIARSRG** **IPIY** **R** **NGSRLT** **KSELEKKIKRSK**
M3—R76D	**SSMHSGMLYKDMVNIARSRG** **IPIYQNGSRLT** **KSELEKKIK** **D** **SK**
M4—S77R	**SSMHSGMLYKDMVNIARSRG** **IPIYQNGSRLT** **KSELEKKIKR** **R** **K**
M5—V48D,L70D,I74D	**SSMHSGMLYKDM** **D** **NIARSRG** **IPIYQNGSRLT** **KSE** **D** **EKK** **D** **KRSK**
M6—K72A,R76A,K78A	**SSMHSGMLYKDMVNIARSRG** **IPIYQNGSRLT** **KSELE** **A** **KIK** **A** **S** **A**
M7—T66A,K67A,S68A	**SSMHSGMLYKDMVNIARSRG** **IPIYQNGSRL** **AAA** **ELEKKIKRSK**
M8—S36D,S37D,S40D	**DD** **MH** **D** **GMLYKDMVNIARSRG** **IPIYQNGSRLT** **KSELEKKIKRSK**

## Data Availability

The data presented in this study are available upon request from the corresponding author.

## Supplementary Materials

The following supporting information can be downloaded at: www.mdpi.com/article/10.3390/v14112348/s1, Figure S1: Secondary structure prediction of the full-length wt:p10 protein determined with PSIPRED [43]; Figure S2: RMSD plots of the c-alpha atoms of the 43 a.a. wt:p10 model (A), and of the 78 a.a. wt:p10 model (B and C), calculated from MD simulations. In B, the RMSD of C-alpha atoms from the 43 a.a. of the 78 a.a. wt:p10 model, and of the full 78 a.a. wt:p10 model (C), are shown. For each model, all three replicate simulation RMSD profiles are colored differently; Figure S3: Histogram of the contact surface area of the equilibrated part of the different simulations of wt:p10 with dsDNA; Figure S4: Primary sequence alignment of the different P10 variants encoded in naturally occurring ASFV isolates; Figure S5: Contact surface area fluctuations of the different p10 mutants with dsDNA throughout the simulation time in all replicate simulations.
