# Supplementary material for: In Silico Characterization of African Swine Fever Virus Nucleoprotein p10 Interaction with DNA
Source: Viruses. 2022 Oct 25;14(11):2348. doi: 10.3390/v14112348 (PMC9694697; doi:10.3390/v14112348)
Supplement: Supplementary file 1 [file viruses-14-02348-s001.zip › viruses-1948743-supplementary.pdf]

**Supplementary Materials**

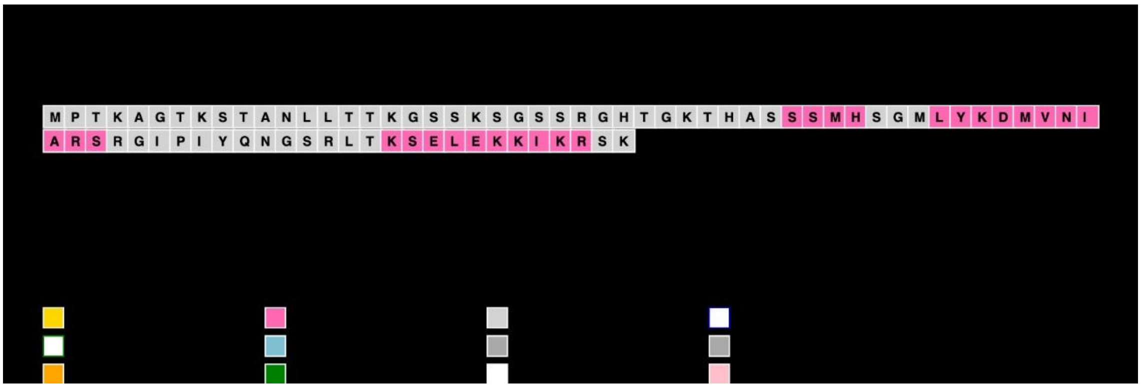


Figure S1. Secondary structure prediction of the full-length wt:p10 protein determined with PSIPRED [43].

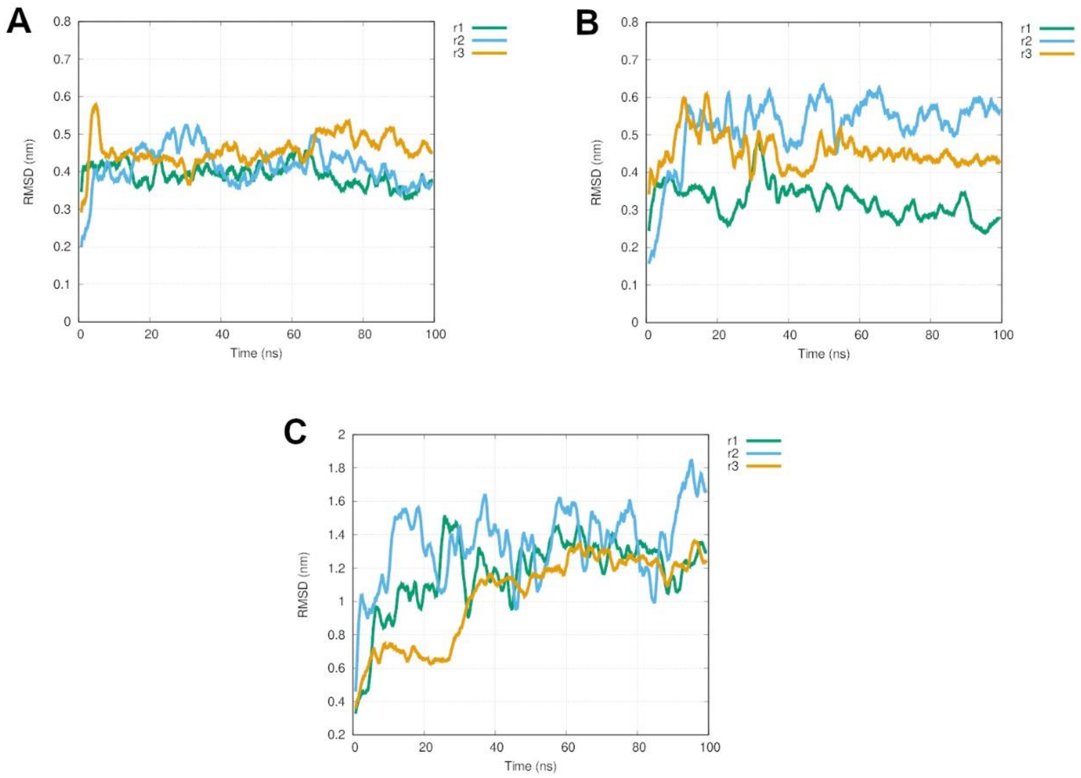


Figure S2. RMSD plots of the c-alpha atoms of the 43 a.a wt:p10 model (A), and of the 78 a.a. wt:p10 model (B and C), calculated from MD simulations. In B, the RMSD of C-alpha atoms from the 43 a.a. of the 78 a.a wt:p10 model, and of the full 78 a.a wt:p10 model (C), are shown. For each model, all three replicate simulation RMSD profiles are colored differently.

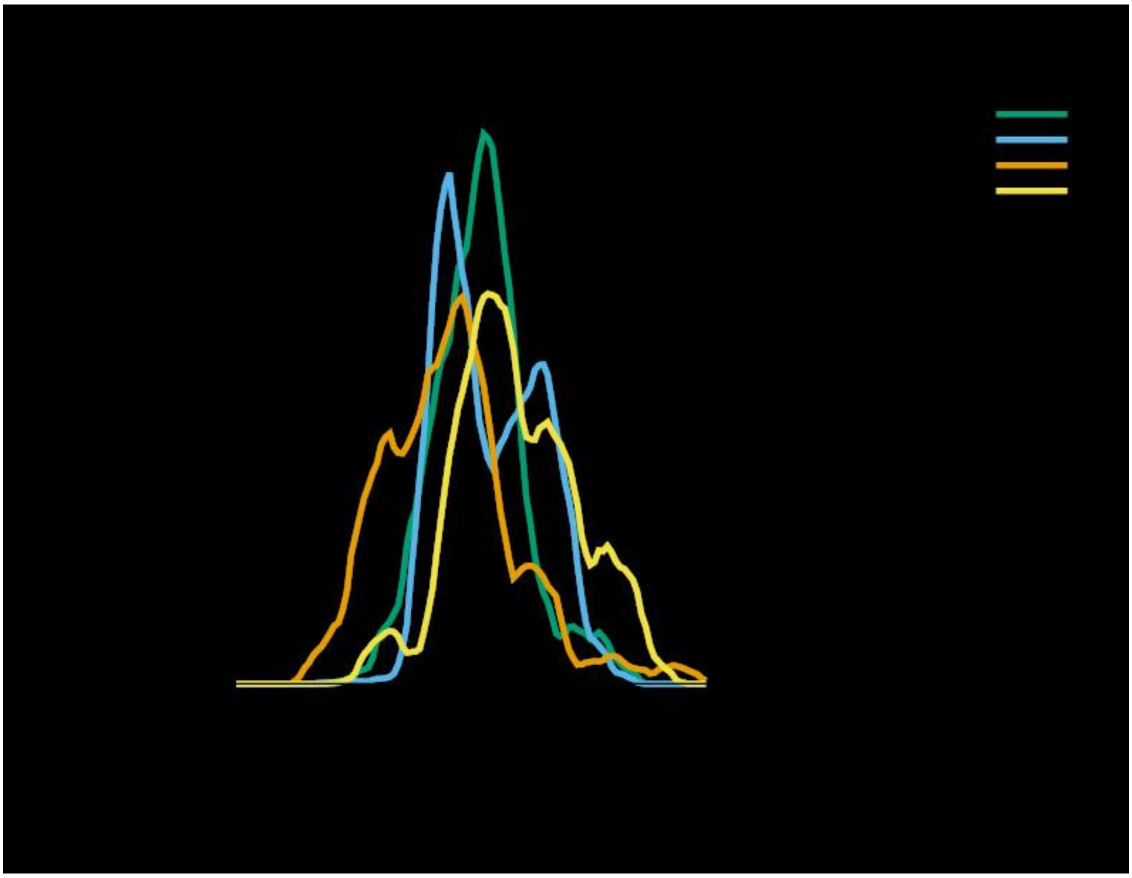

Figure S3. Histogram of the contact surface area of the equilibrated part of the different simulations of wt:p10 with dsDNA.

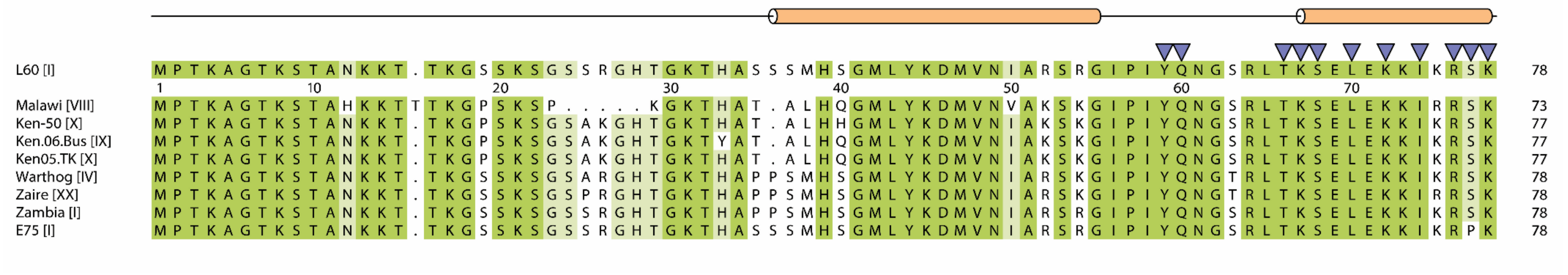


Figure S4. Primary sequence alignment of the different P10 variants encoded in naturally occurring ASFV isolates, colored from white to green according to similarity. The predicted P10 secondary structure is depicted in orange above the alignment. The residues targeted to produce the mutated variants used in the present work are marked with blue arrows. These targeted residues are completely conserved in all wild type variants, except for serine 77, which was replaced by a proline in the E75 strain, suggesting that the produced mutations are not likely to occur spontaneously. Nine unique variants were found using the Basic Local Alignment Search Tool server. Protein accession numbers and, for each case, the number of identical protein entries found (indicated between brackets) are as follows: L60 – NC_044941.1 (149 protein entries, including many from genoptype II); Malawi - P0C9X8.1 (1); Ken-50 – P0C9X7.1 (1); Ken-06.Bus – YP_009702948.1 (2); Ken05.TK – YP_009702783.1 (6); Warthog – P0C9Y0.1 (2); Zaire – QII88571 (5); Zambia – QGM12711 (2); E75 – YP_009703818.1 (2).

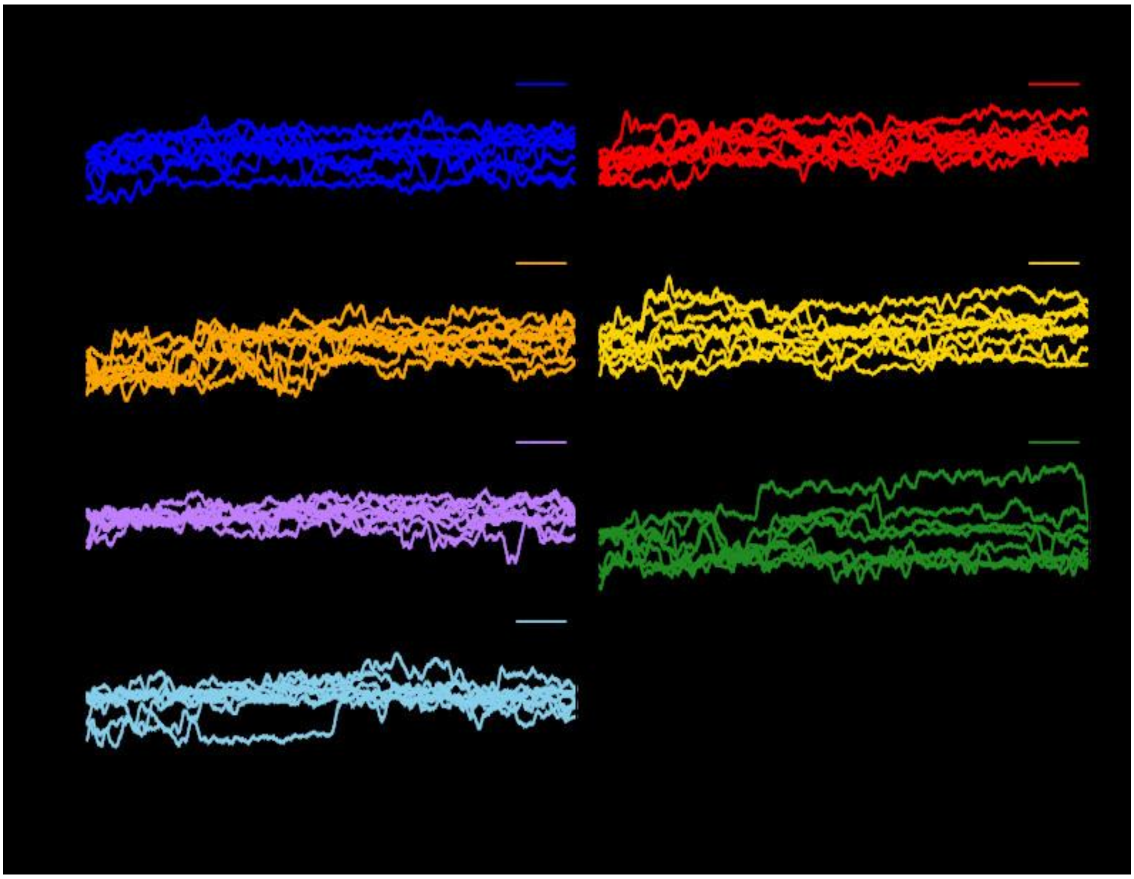

Figure S5. Contact surface area fluctuations of the different p10 mutants with dsDNA throughout the simulation time in all replicate simulations.
